# How Regional Integration Affects Urban Green Development Efficiency: Evidence from Urban Agglomeration in the Middle Reaches of the Yangtze River

**DOI:** 10.3390/ijerph19137937

**Published:** 2022-06-28

**Authors:** Zhen Wang, Xupeng Zhang, Chaozheng Zhang, Qing Yang

**Affiliations:** 1 Institute of Politics and Law, Shihezi University, Shihezi 832003, China; wangzhen102@shzu.edu.cn; 2 College of Public Administration, China University of Geosciences (Wuhan), Wuhan 430074, China; zhangxupeng@cug.edu.cn; 3 College of Public Administration, Huazhong Agricultural University, Wuhan 430074, China; 4 College of Management, Hainan University, Haikou 570228, China; qingyang@hainanu.edu.cn

**Keywords:** regional integration, urban green development efficiency, influence mechanism, urban agglomeration in the middle reaches of the Yangtze River

## Abstract

Unlocking the relationship between regional integration and urban green development efficiency (UGDE) is of great importance for boosting regional high-quality development and promoting sustainable urban development patterns. Although studies have analyzed the spatio-temporal evolution and influencing factors of regional integration and UGDE, the impact of regional integration on UGDE remains untested. In this paper, we construct a conceptual framework to analyze how regional integration can influence UGDE through promoting the factors mobility and optimizing the industrial layout. In addition, we further choose the urban agglomeration in the middle reaches of the Yangtze River (UAMRYR), a rapidly growing urban agglomeration in central China, as a case to investigate the spatial spillover effect of regional integration on UGDE from 2003 to 2017. We quantify the UGDE with a random forest model, then estimate the underlying determinants of the UGDE with a spatial Durbin model. Results indicated that (1) the regional integration level and the UGDE index of the UAMRYR and its three sub-urban agglomerations show an increasing trend; (2) for every 1% increase in the level of regional integration, the level of UGDE will increase by 0.8307%; (3) the impact of regional integration on UGDE has obvious regional heterogeneity; while playing a promoting effect in the Wuhan urban agglomeration and the Changsha-Zhuzhou-Xiangtan urban agglomeration, it shows an inhibitory effect in the Poyang Lake urban agglomeration. We conclude that regional integration in agglomeration areas can accelerate the factors flow and optimize the industrial layout for improving UGDE.

## 1. Introduction

A prominent contradiction is that the carrying capacity of resources and environment is approaching its limit, and the traditional development model with high resource consumption and severe environmental pollution is now no longer sustainable [1]. Green development is a regional sustainable development model, characterized by resources saving and environment protection as its endogenous force, and high-quality economic growth as its driving mechanism [1,2]. Green development has become a key component of its new vision of innovative, coordinated, green, open and shared development. Urban as the basic carrier and objective witnesses of green development, the differences in its geographic location, resource endowment, and ecological environment will directly affect urban green development efficiency (UGDE) [3]. Regional integration, as an advanced spatial organization form of urbanization, is significantly different from the isolated development mode in terms of factor allocation and resource utilization [4]. Therefore, regional integration will inevitably lead to great changes in the mode of urban development. Theoretically, the scale, speed, and degree of regional integration will affect the exchange and transformation, integration, and interaction of economic, social, and ecological elements in the original urban system [5,6,7]. Ultimately, the degree, mode, and efficiency of urban green development will be changed. On the one hand, regional integration can accelerate the factors flow and optimize the industrial layout. This, in turn, will generate different energy consumption and pollutant emission requirements, thereby changing the UGDE [8,9,10,11]. On the other hand, changes in government governance behaviors in regional integration, such as pollution control and urban planning [9,12,13], will help improve the UGDE.

Although scholars have conducted extensive and in-depth research on regional integration and UGDE, the relationship between regional integration and UGDE remains unlocked. Previous research mainly includes the following three aspects: (1) Exploring the impact of industrial agglomeration on urban land use efficiency in the process of regional integration, which was conducted from the perspective of urban economic agglomeration and government behavior [6,14,15,16]. Ullah et al. [13] posited that an increase in the returns to scale induced by agglomeration increases industrial productivity, through both the diversification effect and the specialization effect. Zhang and Zhang [17] studied the influence of industrial agglomeration by a spatial panel model based on Landsat-TM/ETM remote sensing image data and socioeconomic data of Chinese cities at prefecture level and above. Some scholars point out that changes in the flow of capital, technology, innovation, and other factors, and the transfer of industry caused by regional integration, will cause the spatial distribution of economic activities to tend to become scattered or concentrated [6,14]. This, in turn, will increase or reduce the level of UGDE. (2) Quantifying the UGDE of specific urban agglomerations. This research has tried to use multi-factor comprehensive evaluation models, slacks-based measures, etc., to calculate the UGDE, and then use kernel density estimation, space Markov chain, etc., to analyze the temporal evolution, spatial correlation, and influencing factors of the UGDE [3,17,18]. (3) Analyzing the impact of regional agglomeration on specific external performance of UGDE. These studies focus on revealing the internal relationship between regional integration and urban development quality, pollutant emission intensity, and environmental quality convergence [19,20,21,22]. For example, Tanzania promotes the development of the green economy from the perspective of animal husbandry investment and environmental protection [23]. Some scholars have found that Chinese GEE has a significant spatial correlation [24] and technological innovation has a spillover effect [25]. However, the connotations of regional integration and UGDE are very ample; both are multi-dimensional and complex conceptual systems. The above studies have indirectly identified the impact of regional integration on UGDE, but have not directly explored the theoretical and empirical relationship between the two. This paper thus constructs a conceptual framework to analyze how regional integration can influence UGDE through accelerating the factors mobility and optimizing the industrial layout; then we choose the urban agglomeration in the middle reaches of the Yangtze River (UAMRYR), a rapidly growing urban agglomeration in central China, as a case to evaluate the effect of regional integration on UGDE from 2003 to 2017.

The remainder of the paper is structured as follows. In Section 2, the theoretical interaction mechanism between regional integration and UGDE is discussed. After that, the study area, empirical models, and data sources can be found in Section 3. Section 4 presents and discusses the empirical analysis. Finally, the main conclusions, policy implications, and future prospects can be found in Section 5.

## 2. Theoretical Framework

**Figure 1:**
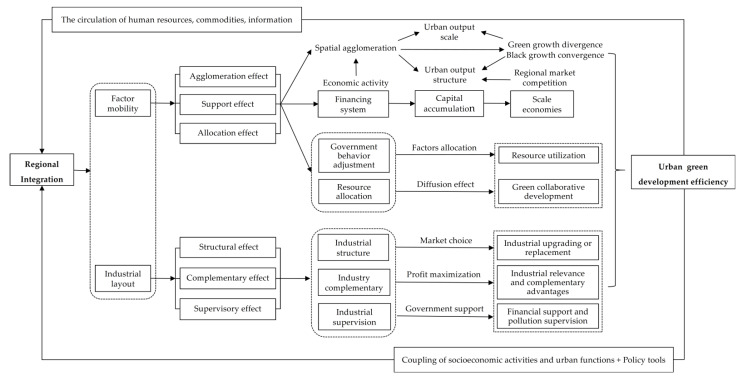
Interaction mechanism between regional integration and the UGDE.

The connotation of regional integration is extremely complex, involving politics, economy, society, ecology, and culture [6,8,26]. As an advanced evolution type of spatial organization form, regional integration mainly takes factor integration and industrial integration as the core process and key link. This means that, no matter what aspect of the integration process is involved, it will end up in the allocation scale and structure of various natural and unnatural production factors and the spatial agglomeration between different industries [6,11]. Ultimately, the UGDE will be affected. On the contrary, various policies and regulations promulgated around urban green development will also improve the level of regional integration. Specifically, the impact of regional integration on the UGDE is mainly reflected in the following aspects (Figure 1):

(1) Factor mobility: first is the agglomeration effect. Regional integration is not only an important driving force behind the promotion of the spatial agglomeration of regional economic activities, but is also a key driver that will change the scale of urban output and the reconstruction of spatial structure [4]. Driven by regional market competitiveness, the enhancement of factor mobility will promote the formation of integrated and specialized spatial agglomerations of clean and polluting production factors in different geographical units. This will promote the growth convergence and pollution of urban clean output; the reduction in output converges and ultimately improves the efficiency of urban green output [5,14]. Second is the support effect. Regional integration can use financing mechanisms to convert various factors of production into capital, promote the accumulation of regional capital, and support the joint creation, investment operations, and the technology research and development of various industries [27,28]. Under the background whereby micro-entities obtain more capital factor investment, the supporting effect produced by the increase of capital investment scale in this process can stimulate the flow of factors to production fields with high-quality investment returns through a variety of transmission paths. Examples of such paths include technological diffusion, human compensation, and information sharing, as well as the formation of economies of scale, all of which can promote the improvement of UGDE. Third is the allocation effect. The distribution pattern of factors between different regions, periods, and subjects directly affects the level of urban green development [29]. As regional integration has advanced, government behavior has shifted from competition to cooperation, which has promoted more limited resources to be invested in regions or subjects with higher productivity and output growth rates. Then, factor allocation efficiency improves, along with the resource utilization efficiency and green development level of the region and the whole of society. At the same time, the diffusion effect formed between high allocation efficiency and low allocation efficiency causes the two to tend to check and balance each other. This may further lead to a joint increase in the UGDE in different regions.

(2) Industrial layout: first are the structural effects. Regional integration has strengthened the spatial relationship between cities, and the polarization phenomenon produced by the “center + periphery” structure is gradually replaced by the trickle-down effect between regions [25]. To obtain optimal externality, the industrial structure is gradually upgraded. At this time, urban industrial activities and economic production enter a period of structural adjustment. On the one hand, the development of regional integration, information, and services causes the economic service industry to have a stronger competitive advantage under the background of market choice. The upgrading of industrial structure is conducive to the optimization of urban economic output structure and green production [16]. On the other hand, the weakening of trade barriers reduces communication costs and cooperation scope among industries. In this process, polluting industries with low competitive efficiency are replaced, and the “economic service” trend of industrial structure is more prominent; finally, the green development of cities is improved. Second is the complementary effects. Driven by the realization of the maximization of economy and income within the scope of regional integration, industries tend to congregate in a certain geographic space. They form an industrial community or industrial production chain that is interconnected, and the industries promote and complement each other. When such a community or industrial chain develops to a certain scale, it will have a diffusion–radiation effect on adjacent areas. Coupled with the spatial spillover effect of the industry’s technical knowledge, the complementarity of the two effects will affect the UGDE to a certain extent. Third is the supervision effects. The continuous improvement of the level of regional integration will directly or indirectly encourage local governments to further strengthen the supervision, management, and control of urban industries. This will prompt micro-entities to improve production performance, and thus promote economic green growth and environmental protection [30,31]. Regional integration can strengthen the supervision of urban green development in two ways. On the one hand, regional integration is conducive to industrial integration and professional development. This integration process itself can prompt more external investors to supervise industrial operational efficiency and environmental costs. On the other hand, the strengthening of formal government supervision and informal public supervision in the regional integration process can not only provide more financial support for urban environmental pollution control, but can also provide more R&D funds for environmental protection production technology. In addition, increased supervision can also provide priority policy support to the environmental protection industry and new energy industry, and ultimately, improve the level of the UGDE.

In addition, changes in the UGDE will also adversely affect regional integrated development. First of all, urban green development is one of the core contents of regional integrated development. In areas with high levels of urban green development, such development can have a strong reverse promotion effect on economic, social, and ecological construction and transformation, thereby accelerating the process of regional integration [32]. Secondly, urban green development is the external thrust behind regional integration. On the one hand, the economic agglomeration, infrastructure agglomeration, and the horizontal and vertical expansion of the industrial chain formed with the optimization and upgrading of the industrial structure will promote the extension of the regional spatial scope. This will facilitate the organic combination of socio-economic development and urban functions in the process of regional integration [33]. On the other hand, the plans and policies formed around the green development of cities are important policy tools that can be used to promote regional integrated development.

## 3. Materials and Methods

### 3.1. Study Area

**Figure 2:**
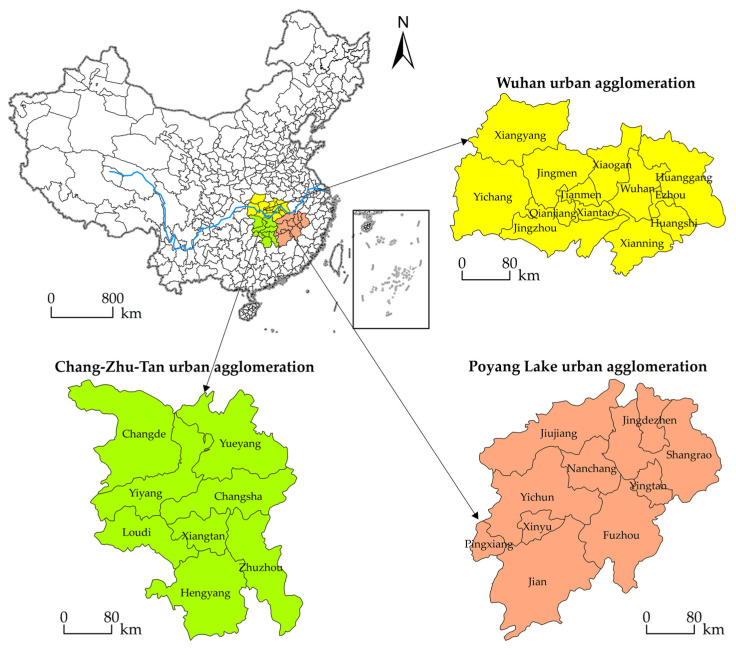
Location and range of the UAMRYR and its sub-urban agglomerations.

The UAMRYR is located in the central part of China, which was formed by 31 cities of Hubei, Hunan, and Jiangxi Province. It consists of the three largest national-level urban agglomerations in China by embracing the Wuhan urban agglomeration, the Changsha-Zhuzhou-Xiangtan (Chang-Zhu-Tan) urban agglomeration, and the Poyang Lake urban agglomeration (Figure 2). The UAMRYR extends over an area of 317,000 km^2^, equivalent to 3.3% of the national territory, but it accounts for 9.7% of the country’s total population and 9.6% of the country’s total economic output as of 2017 [34]. With the implementation of the “Development Plan for the UAMRYR”, it has actively responded to the national regional integration policy and has made substantial progress. However, the UAMRYR is still in the primary stage of development, problems such as high environmental pollution, severe ecosystem degradation, tightening resources constraints, and extensive industrial development have become increasingly prominent [35]. Under such a realistic background, how to accelerate the integration process while improving the UGDE has become more and more urgent and important.

### 3.2. Empirical Method

#### 3.2.1. Random Forest Method for Measuring UGDE

Random forest method (RF) is a machine learning algorithm proposed by Leo, which uses the decision tree classifier to make a comprehensive classification [36]. RF has the advantage of strong data mining capabilities and high prediction accuracy; it can achieve high classification accuracy with optimal parameters and minimum errors based on limited training datasets and establish a weight learning mechanism between multiple indicators, thereby solving the problem of “over-fitting” of a certain attribute in a complex and nonlinear large system [37,38]. Urban green development is a complex system with highly interactive coupling between social economy and ecological environment, and the UGDE has the characteristics of complexity, non-structural and random uncertainty. Therefore, a more robust and flexible method is needed to deal with nonlinear relationships, high-order correlations, and even missing values. Practically, RF has been widely applied to measure the urban land use efficiency, evaluate the urban development quality, and predict the regional development trends. We attempt to introduce the RF into the evaluation of UGDE. In this study, we use Stata software applications to construct RF. The process can be summarized as follows: new training datasets are generated by conducting the bootstrap method on the original training datasets; a regression tree is grown for each new training dataset; each regression tree generates one predicted value, and the mean value of them is the UGDE. In addition, two-thirds of the data in the original datasets are selected randomly to generate a training dataset, while the other one-third are used to form the corresponding testing datasets to have a sample as representative as possible. The construction of the RF model is as follows:

First, construction of the classification tree. The bagging algorithm is introduced into the cart decision tree for multiple random sampling, then a single decision tree classifier based on the *Gini* index is trained. The *Gini* index of node *n* is defined as $G\left(n\right)=\sum_{i\neq j}p\left(w_{i}\right)p\left(w_{j}\right)=1-p^{2}\left(w_{j}\right)$, where $p\left(w_{i}\right)$ is the frequency of the number of class I samples in the number of training samples on node *n*.

Second, RF weight calculation. The specific implementation steps are as follows:

(1) $$\begin{array}{l} \Delta_{j}=\left|\sum_{j=1}^{N}\left(Gini_{e_{i}}-Gini_{e_{i}^{j}}\right)/N\right|\\ RFW_{j}=\Delta_{j}/\sum_{j=1}^{N}\Delta_{j} \end{array}$$

where $\Delta_{j}$ denotes the reduced value of *Gini*, *N* denotes the number of indicators, *RFW_j_* denotes the weight value of the *j*th decomposed indicator.

Third, weighted combination. Outputing the RF weight of each decomposition variable, one generates the optimal simulation relationship between each index variable and UGDE according to the classification tree linear mapping rule.

#### 3.2.2. Spatial Durbin Model

Complicated economic, social, and environmental linkages are involved among cities, which leads to a spatial spillover effect in urban development [15]. That is to say, the sample data that we collected does not necessarily conform to the premise assumption of being independent and identically distributed, and continuing to use the traditional econometric model will inevitably lead to biased results. However, the traditional econometric models like the ordinary least squares (OLS) model did not consider the spatial effect of the explanatory variable and explained variable. Therefore, we constructed a spatial econometric model to investigate the spatial spillover effect of regional integration on UGDE. Current spatial econometric models mainly include the spatial lag model (SLM), spatial error model (SEM), and spatial Dobbin model (SDM). These three models, respectively, consider the spatial dependence and spillover characteristics of independent variable spatial lag term, dependent variable spatial lag term and spatial lag term of both independent variables and dependent variables. The three models have excellent explanatory power in investigating spatial effects. According to LeSage and Pace [39] and Elhorst [40], the SDM is more acceptable than both the spatial autoregressive (SAR) model and the spatial error model (SEM), and thus should be given priority in empirical analysis. In the same vein as LeSage and Pace [39], we construct the baseline regression model as follows:

(2) $${\mathrm{UGDE}}_{\mathrm{it}}={\alpha I}_{N}+{\rho \mathrm{WUGDE}}_{\mathrm{it}}+{\beta \mathrm{RI}}_{\mathrm{it}}+{\gamma X}_{\mathrm{it}}+{\theta \mathrm{WRI}}_{\mathrm{it}}+\pi {\mathrm{WX}}_{\mathrm{it}}+\varepsilon_{\mathrm{it}}$$

where *i* and *t* denote city and year, respectively; *UGDE* denotes urban green development efficiency; *RI* denotes regional integration level; *X* denotes control variables; *W* denotes spatial weight matrix. The geographical adjacency matrix is used in this study. Specifically, it is considered that if two areas are adjacent, the value is assigned as 1, and if they are not adjacent, the value is assigned as 0. Based on this, this paper establishes a matrix whose main diagonal elements are all 0, and then standardizes the elements of each row according to the usual practice to obtain the spatial matrix. *a* represents constant item; *I_N_* represents unit matrix; *ρ*, *θ*, *π*, and *β* represent the parameter to be estimated.

### 3.3. Variable Selection

#### 3.3.1. Explained Variable: UGDE

The methods used to measure the level of regional green development or urban development quality can be classified into two categories: efficiency method and index method [1,2,17]. The efficiency method mainly uses the Data Envelopment Analysis (DEA) model and the Slack Based Measurement (SBM) model to measure the input–output efficiency, emphasizing that it can achieve higher economic output with less resource input while simultaneously reducing environmental pollution. Although the efficiency method can emphasize the core content of green development, they do not strictly distinguish green development from ecological efficiency, and this may lead to biased results. The index method mainly measures the level of UGDE by constructing a multi-dimensional indicators system. Compared with the efficiency method, the index method can better reflect the essential characteristics of green development and highlight the complexity, particularity, and dynamic quality of the UGDE [1,4,8]. On the basis of the research of Liu et al. [2] and Zhang and Zhang [17], 14 indicators from three aspects of scale, agglomeration, and benefit are selected to calculate the UGDE (Table 1).

#### 3.3.2. Explanatory Variable: Regional Integration

A unified conceptual framework, or a comprehensive evaluation index system for regional integration, has not been yet formed, and the calculation of the level of regional integration is also still in the preliminary exploration stage. Most scholars have only focused on the specific external performance of regional integration, and they have measured the level of regional integration by calculating economic integration, market integration, spatial integration, or transportation integration [4,6,7,8,9,10]. In fact, as a special form of spatial and regional evolution, regional integration is also a process of horizontal development and in-depth promotion. On the basis of existing research in various fields, a total of eleven indicators were selected from the four aspects of economic, market, spatial, and administrative integration. These indicators were then used to build a comprehensive measurement index system of regional integration (Table 2). We introduce the entropy method to calculate the level of regional integration, and the process can be summarized as follows: Because the individual indicators have inconsistent scales or distributions, we normalized the data by the minimum–maximum standardization method; we use the entropy eight method to determine the weight of each evaluation indicator; according to the normalized value and weight of each indicator, we employ the weighted index method calculated the level of regional integration.

#### 3.3.3. Control Variables

In addition to regional integration, other factors such as urbanization level, industrial structure, and technological level will exert an influence on UGDE. Therefore, the influence of some important factors should be controlled, to obtain more accurate results. The proportion of the urban population to the total population, the ratio of the output value of the tertiary industry to the output value of the secondary industry, the proportion of oil energy in the total energy consumption, the actual use of foreign direct investment in the region, and the investment in scientific research funds are selected as control variables. These variables can reflect the impact of urbanization level (UL), industrial structure (IS), energy structure (ES), technological level (TL), and economic openness (EO) on UGDE, respectively.

### 3.4. Data Source

The data are obtained from the *China Urban Statistical Yearbook (2004–2018)*, *China Statistical Yearbook (2004–2018)*, and *China Environmental Statistics Annual Report (2004–2018)*. However, some dates were missing in the above yearbooks. Therefore, we collected information from the statistical yearbooks and bulletins of provinces and cities. Some missing values are supplemented by the linear interpolation method. Considerations of GDP and fixed asset investment have been converted into the 2003 constant price.

## 4. Results and Discussion

### 4.1. The Spatio-Temporal Evolution of Regional Integration and the UGDE

**Figure 3:**
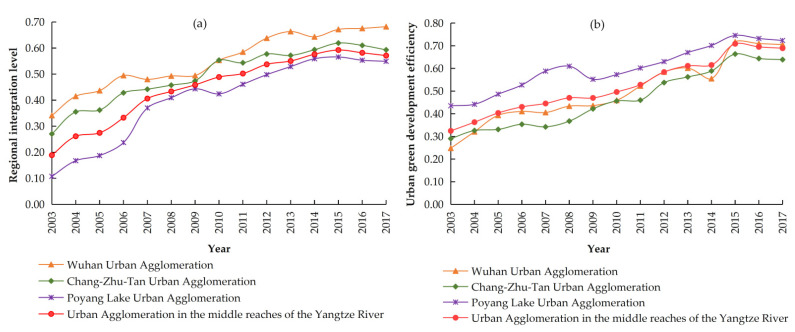
Spatio-temporal evolution of the regional integration level and the UGDE. (**a**) Regional integration leve; (**b**) UGDE.

Figure 3 shows the spatio-temporal evolution of the regional integration level and the UGDE index of the UAMRYR and its sub-urban agglomerations from 2003 to 2017. In terms of the absolute value change of regional integration, the overall regional integration level of the UAMRYR shows a trend of increasing year by year, increased from 0.2398 in 2003 to 0.6082 in 2017, with an average annual growth rate of 12.80% At the same time, the regional integration level of each sub-urban agglomeration also shows a growth trend at different magnitudes. Among them, the absolute value of the regional integration level of the Poyang Lake urban agglomeration increased the most, from 0.1077 in 2003 to 0.5492 in 2017, with a net increase of 0.4415. The absolute value of the regional integration level of the Wuhan urban agglomeration increased from 0.3412 in 2003 to 0.6821 in 2017, with a net increase of 0.3409. The absolute value of the regional integration level of the Chang–Zhu–Tan urban agglomeration increased the least, from 0.2704 in 2003 to 0.5932 in 2017, with a net increase of 0.3228. However, the average annual growth rate, from high to low, were: the Poyang Lake urban agglomeration, 34.16%; the Chang-Zhu-Tan urban agglomeration, 9.95%; and the Wuhan urban agglomeration, 8.33%.

In terms of the development of the UGDE, although regional differences persisted, the UGDE in the UAMRYR and its three sub-urban agglomerations shows a fluctuating increase. For the UAMRYR, its UGDE was increased from 0.3246 in 2003 to 0.6892 in 2017, with an increase rate of 112.34%. In terms of the three sub-urban agglomerations, the absolute value changes, from large to small, were Wuhan urban agglomeration (0.4573), Chang-Zhu-Tan urban agglomeration (0.3486), and Poyang Lake urban agglomeration (0.2880), respectively. However, the average annual growth rate of the UGDE, from large to small, were Chang-Zhu-Tan urban agglomeration (15.36%), Wuhan urban agglomeration (10.01%), and Poyang Lake urban agglomeration (5.51%), respectively. Similar to the results of Liu et al. [2], from 2003 to 2017, the UGDE in China showed a positive spatial correlation. The Wuhan urban agglomeration benefited from early policy preferences, which meant that urban green development in Wuhan urban agglomeration was far ahead of other regions.

### 4.2. Selection of Relevant Test and Measurement Models

The ADF unit root and cointegration tests of UGDE, regional integration, and control variables were carried out, and the results show that its difference sequence were first-order single-integration. Therefore, there was a long-term and stable cointegration relationship between variables, which met the conditions for the econometric test. Table 3 reports the OLS and SDW regression results. The robust LM test statistics of the two models were both significant at the 5% level, indicating the existence of significant spatial auto-correlation and that a spatial econometric model should be adopted. Secondly, to further determine the specific form of the spatial panel model, Wald statistics were used to test whether the SDM could be weakened into an SLM or SEM model. In order to facilitate the comparison of results, we give the OLS regression results based on fixed effect. According to the test results, the R^2^ of the SDM model improved to 0.8203, which is much greater than the 0.6597 of the OLS model. In addition, the Wald statistics were significant at the statistical level of 10%. Further, combined with the results of the Hausman test, a generalized SDM model based on fixed effects was selected.

### 4.3. Decomposition of UGDE Effect of Regional Integration and Its Chronological Evolution

The above research results verify the fact that the regional integration of a region will not affect the UGDE of the region itself, but also the UGDE of its adjacent regions. That is, regional integration has both a direct and indirect impact on UGDE. Therefore, this study further examines the average spillover effect of regional integration on UGDE. Table 4 reports the direct, indirect, and total effect of regional integration on UGDE. For every 1% increase in the level of regional integration, the level of UGDE will increase by 0.8307%, including a 0.7088% increase in the direct effect and a 0.1217% increase in the indirect effect.

From 2003 to 2019, the direct effects of regional integration on UGDE were significantly positive. This implies that increasing the scale and potential capacity of capital support for regional integration would help in improving the UGDE. This in turn suggests that the agglomeration, support, structure, complementary, and supervision effects of regional integration played a positive role during this period. The impact of urbanization level, energy structure, and economic opening level on the UGDE is positive, but the promotion effect of the urbanization level did not pass the significant test. In contrast, the effect of industrial structure on UGDE has not been formed. The reason may be that the UAMRYR is an old industrial base in China. The proportion of traditional industries in the whole system remains high, and due to the large state-owned components of tertiary service industries (such as telecommunications and finance), the government’s capital monopoly restricts the development of private enterprises. These limit the positive effect of the rationalization and upgrading of the industrial structure on UGDE. At the same time, due to the differences in the development of Hunan, Hubei, and Jiangxi Provinces, as well as the high degree of industrial overlap, how to break the regional blockade and interest barriers, promote the factors flow, and optimize the industrial structure has become the key to improving the UGDE. In terms of indirect effects, for every 1% increase in the level of regional integration, the UGDE increased by 0.0063%. This finding indicates that regional integration has a positive spatial spillover effect on UGDE in surrounding areas. From 2010 to 2017, the direct and indirect effects of regional integration on UGDE both increased. The two effects were also significant at the statistical level of 1% and 10%, respectively, and the total effect increased to 0.9805, indicating that further promoting regional integration can accelerate the transformation of urban development from “black” to “green”. From the perspective of control variables, with time passing by, the direct effect of the urbanization level on UGDE gradually became prominent, and the total effect increased. The coefficient of industrial structure and technical level changed from negative to positive, but the promotion effect of industrial structure’s spatial spillover effect on UGDE has not yet been formed. The role of energy structure and economic openness in promoting the UGDE gradually increased, and both passed significant level tests at different levels (10% and 5%, respectively). During the development of green economy, the dependence on natural resources was reduced by technological innovation, and this had a significant inhibition effect on UGDE.

### 4.4. Regional Differences in Urban Green Development Effects of Regional Integration

Since the development levels of regional integration within the UAMRYR are not synchronized, significant regional differences exist in the UGDE effect of regional integration (Table 5). From the perspective of the direct effect, the influence of regional integration on UGDE is in the order of: Wuhan urban agglomeration > Chang-Zhu-Tan urban agglomeration > Poyang Lake urban agglomeration. From the perspective of the indirect effect, Wuhan urban Agglomeration > Poyang Lake urban agglomeration > Chang-Zhu-Tan urban agglomeration. From the perspective of the total effect, Chang-Zhu-Tan urban agglomeration > Poyang Lake urban agglomeration > Wuhan urban agglomeration. These results show that among the three urban agglomerations within the UAMRYR, the Wuhan urban agglomeration has the highest integration level, and the strongest ability to accept the spatial spillover effects of capital, information, and technology in the surrounding areas, and the strongest role in promoting the level of UGDE. In contrast, the regional integration of Poyang Lake urban agglomeration is still in its infancy. The role of its regional integration and the regional integration of the surrounding urban agglomerations in improving the UGDE has not been fully highlighted. As can be seen, the lack of inter-regional cooperation and unified planning may block the spatial transmission channel of the regional integration level and become an obstacle to the increase of UGDE. In the future, attention should be focused on enhancing economic and technological connections between cities and boosting the flow of capital across regions. In addition, the influence of each control variable on UGDE also shows obvious regional heterogeneity. For example, the control variable that played a leading role in improving the UGDE of the Wuhan urban agglomeration was the energy structure, but was the industrial structure of the Chang-Zhu-Tan urban agglomeration, and was the energy structure of the Poyang Lake urban conglomeration.

### 4.5. Robustness Test

Since regional integration is a long-term transformation process, the impact of capital flow and factor accumulation in the process of regional integration on the UGDE may not appear in the current period. Therefore, to more accurately obtain and grasp the impact of regional integration on UGDE, regional integration is further lagged for one period before SDM regression (Table 6). The comparison shows that the coefficient direction and significance of the main variables have little change, indicating that the conclusions are robust. It is worth noting that the impact of regional integration on UGDE will weaken over time. This means that regional integration is bound to be a major strategy to which the UAMRYR will adhere and further promote for a long time.

## 5. Implications

### 5.1. Policy Implications

The conclusion of this paper has the following implications. (1) Boosting the development of regional integration in the UAMRYR is an effective means to improve the UGDE. Firstly: accelerating the construction of the planning and implementation system of key functional areas; taking the functional areas as the implementation subject, to establish the regional development policy system of “multi-level control, classified guidance and accurate implementation”, and consistent with the functions of administrative subjects at all levels. These should be done according to the functional status and development orientation of various regional subjects. Secondly: promoting the free flow and optimal allocation of production factors in the process of regional integration. Form an integrated market resource allocation mechanism, such as an industrial chain, supply chain, and capital chain, to improve the collaborative development efficiency and potential among urban agglomerations. This will further accelerate the transformation and upgrading of the urban development model. (2) Exploring differentiated paths of urban green development in the process of regional integration. In general, based on the principle of regional balanced development, a mechanism for integrating multiple elements such as economy, market, space, and administration should be constructed, to narrow the regional heterogeneity in the impact of regional integration on UGDE. The Wuhan urban agglomeration should focus on the integration of traditional industries and high-tech industries, strengthen industrial technological transformation and model innovation. Further, they should promote the optimization and upgrading of traditional industries, accelerate the development of the modern service industry, and form a new driving force to support urban green development. The Chang-Zhu-Tan urban agglomeration should optimize the spatial linkage environment, eliminate internal city administrative cooperation and factor flow barriers, and give full play to the spatial spillover effect of the regional integration. At the same time, each city must be encouraged to actively utilize its location advantages, improve regional openness, and maximize the overall effect of regional integration on UGDE. The Poyang Lake urban agglomeration should pay attention to the synergy between industrial development and ecological protection. At the same time, provincial capital cities should comprehensively improve their functions of high-end factor agglomeration, scientific and technological innovation, and comprehensive services. Other cities should also improve their abilities in terms of urban green development, while accelerating the process of regional integration.

### 5.2. Future Prospects

This study has several research limitations. First, because of limited availability of socioeconomic data, only 2 unexpected output indicators are used to measure UGDE. Second, because of space reasons, a case study is used as supporting evidence rather than an in-depth and large-scale study. However, the case study method can help readers to better understand the research questions and refine the presented research methods and conclusions drawn in this study. Therefore, with improvements and further study, the timescale of the study could be broadened to capture the latest changes of UGDE. Moreover, the intermediary path of regional integration affecting UGDE can be further studied. Thirdly, this research does not consider the socioecological conditions, as well as the previous situation of the environmental condition when studying how regional integration can influence UGDE. Therefore, the next research focus is to use the difference-in-differences (DID) model to demonstrate the impact of regional integration policy on UGDE from the perspective of policy.

## 6. Conclusions

In this paper, we construct a conceptual framework to analyze how regional integration can influence UGDE through the factors mobility and industrial layout. In addition, we further choose the UAMRYR as a case to evaluate the effect of regional integration on UGDE. It can provide the theoretical framework and empirical evidence in unlocking the link between regional integration and UGDE. The main conclusions are summarized as follows. (1) Regional integration can significantly improve the UGDE. Regional integration can accelerate the factors flow and optimize the industrial layout, and then produce agglomeration, support, allocation, structural, complementary, and supervision effects, which ultimately improve the UGDE. (2) The total, direct, and indirect effects of regional integration on UGDE are significantly positive. For every 1% increase in the level of regional integration, the level of UGDE will increase by 0.8307%, including a direct effect of 0.7088% and an indirect effect of 0.1217%. (3) The impact of regional integration on the UGDE has obvious regional heterogeneity; while playing a promoting effect in the Wuhan urban agglomeration and Chang-Zhu-Tan urban agglomeration, it shows an inhibitory effect in the Poyang Lake urban agglomeration. A 1% increase in regional integration also increases UGDE by 0.0728% for Wuhan urban agglomeration, 1.0443% for the Chang-Zhu-Tan urban agglomeration, but −0.1472% for the Poyang Lake urban agglomeration.

## Figures and Tables

**Table 1:** Indicators system of UGDE.

Target Layer	Criterion Layer	Index Layer
Scale	Labor input	Total employment at the end of the year
	Capital input	Total investment in fixed assets
	Energy input	Total electricity consumption of the whole society
Agglomeration	Population agglomeration	$C=\frac{1}{2}\sum_{i=1}^{n}\left|X_{i}-Y_{i}\right|$
	Industrial agglomeration	$Gini={\sum_{i}\left(s_{i}-x_{i}\right)}^{2}$
	Urban spatial density	Expansion intensity of built-up area
Benefit	Economic growth	Real GDP per capita
		Total retail sales of consumer goods per land
	Social development	Urban per capita construction land area
		Urban per capita disposable income
	Ecological friendliness	Greening rate of built-up area
		Harmless treatment rate of domestic garbage
	Environmental pollution	Emission intensity of industrial pollutants
		Total carbon emissions

Note: In the calculation of population agglomeration degree, $X_{i}$ represents the proportion of the total population of i to the total population of the urban agglomeration; $Y_{i}$ represents the proportion of the land area of the i city administrative region to the total land area of the urban agglomeration. In the calculation of industrial agglomeration degree, $s_{i}$ denotes the proportion of the number of employed persons in a certain industry to the total number of employed persons; $x_{i}$ denotes the proportion of the total number of regional employment to the total number of economic employment.

**Table 2:** Indicators system of regional integration.

Target Layer	Criterion Layer	Index Layer
Economic integration	Balanced economy development	$RAT=\sum_{i}\left|\frac{Y_{i}/Y}{L_{i}/L}-1\right|$
	Economic openness	Total Imports/Total Exports
	Economic contact intensity	$R_{ij}=\frac{\sqrt{P_{i}V_{i}}\times \sqrt{P_{j}V_{j}}}{D_{ij}}$ $F_{ij}=\frac{R_{ij}}{\sum_{j=1}^{n}R_{ij}}$
Market integration	Pedestrian volume	Regional population flow number
	Commodity flow	Change rate of freight turnover
	Cash flow	Fixed asset investment growth rate
Spatial integration	Traffic accessibility	Highway mileage/Total area
	Information diffusion	Total business volume of post and telecommunications/Total population
	Population offset growth rate	$P=|X_{i}-{\bar{X}}_{i}|$
Administrative integration	Strategic agreement	Number of regional cooperation meetings
	Policy identity	Regional customs clearance integration (0/1)

Note: In the calculation of balanced economy development degree, Y and L denote the output value and the employee number of the corresponding industry; when i is equal to 1, 2, and 3, it represents the primary, secondary, and tertiary industry, respectively. In the Economic contact intensity, P and L denote the total fixed asset investment per area and the employee number in the tertiary industry per area; D denotes distance between regions. In the calculation Population offset growth rate, $X_{i}$ denotes the average annual growth rate of the population of the *i*-th city; ${\bar{X}}_{i}$ denotes the average annual growth rate of the population of urban agglomeration.

**Table 3:** OLS and SDM regression results.

Variable	OLS Model		SDM Model	
*RI*	0.0205 **	(0.0095)	0.0077 *	(0.0044)
*UL*	0.1371 *	(0.0741)	−0.0604	(0.4632)
*IS*	0.0450	(0.0842)	−0.0042 ***	(0.0007)
*ES*	0.6513 ***	(0.2117)	0.2053 **	(0.0935)
*TL*	−0.2702	(1.109)	−0.5492 *	(0.3002)
*EO*	−0.0865	(0.1744)	−0.4054	(0.5191)
*W·RI*	——		0.0601 ***	(0.0059)
*W·UL*	——		0.0842	(0.1077)
*W·IS*	——		0.0355 ***	(0.0039)
*W·ES*	——		0.1041 *	(0.0608)
*W·TL*	——		0.0337	(0.1064)
*W·EO*	——		−0.0037	(0.1018)
*Cons.*	−1.2036 *	(0.6389)	−1.1878 ***	(0.3944)
R^2^	0.6597		0.8203	
LM test no spatial lag	4.4723 **		——	
Robust LM test no spatial lag	9.2997 ***		——	
LM test no spatial error	0.8418 **		——	
Robust LM test no spatial error	4.7884 **		——	
Wald_spatial lag	——		11.4223 **	
Wald_spatial error	——		14.0005 *	
Hausman test probability	——		0.0030	

Note: *, **, *** denote statistical significance at the 10%, 5%, and 1% level, respectively; the standard deviation is given in brackets.

**Table 4:** Decomposition of UGDE effects of regional integration.

Variable	2003–2017			2003–2009			2010–2017		
	Direct Effect	Indirect Effect	Total Effect	Direct Effect	Indirect Effect	Total Effect	Direct Effect	Indirect Effect	Total Effect
*RI*	0.7088 *** (0.0291)	0.1217 * (0.0712)	0.8307 ** (0.3320)	0.6910 *** (0.2308)	0.0063 (0.0133)	0.6973 * (0.3873)	0.8772 *** (0.2077)	0.1033 * (0.0573)	0.9805 * (0.5160)
*UL*	−0.1013 (0.0784)	−0.0043 (0.0339)	−0.1056 (0.0771)	0.1023 (0.9018)	−0.2006 * (0.1055)	−0.0983 (0.1099)	0.1145 ** (0.0673)	0.0053 (0.0337)	0.1198 (0.2113)
*IS*	0.3011 * (0.1780)	−0.0055 (0.0361)	0.2956 * (0.1738)	−0.2313 ** (0.1306)	0.0003 (0.0052)	−0.2313 ** (0.0855)	0.0094 (0.0712)	−0.0125 * (0.0073)	−0.0031 (0.0053)
*ES*	0.4055 *** (0.0179)	0.0043 (0.0187)	0.4098 ** (0.1639)	0.2077 * (0.1153)	−0.0035 (0.1033)	0.2042 * (0.1187)	0.4005 ** (0.1907)	0.0177 * (0.0090)	0.4181 * (0.2201)
*TL*	−0.0928 (0.0431)	−0.0005 (0.0093)	0.0933 (0.0648)	−0.1144 (0.0837)	0.0003 (0.0107)	−0.1141 (0.0553)	0.0073 (0.0044)	0.0127 * (0.0071)	0.0200 (0.0103)
*EO*	0.1012 ** (0.0361)	−0.0043 (0.0493)	0.0969 (0.0868)	0.1012 ** (0.0562)	−0.0003 (0.0019)	0.1009 * (0.0583)	0.2113 *** (0.0177)	0.0104 * (0.0055)	0.2217 ** (0.0923)
*Cons.*	2.0453 *** (0.8492)	−1.0495 *** (0.4458)	0.9490 (0.0749)	−1.5045 *** (0.6392)	0.9324 *** (0.2054)	1.5032 *** (0.6374)	0.7473 *** (0.2055)	−0.9402 * (0.4948)	−0.7492 ** (0.3121)
R^2^	0.3003	0.4117	0.5055	0.2917	0.4812	0.6743	0.7044	0.6824	0.5002

Note: *, **, *** denote statistical significance at the 10%, 5%, and 1% level, respectively; the standard deviation is given in brackets.

**Table 5:** Decomposition of urban green development effects of regional integration.

Variable	Wuhan Urban Agglomeration			Chang-Zhu-Tan Urban Agglomeration			Poyang Lake Urban Agglomeration		
	Direct Effect	Indirect Effect	Total Effect	Direct Effect	Indirect Effect	Total Effect	Direct Effect	Indirect Effect	Total Effect
*RI*	0.0497 *** (0.0219)	0.0231 * (0.0124)	0.0728 *** (0.0270)	1.0305 * (0.5570)	0.0128 * (0.0075)	1.0433 * (0.5491)	0.0250 (0.1104)	−0.0172 (0.0911)	−0.1472 (0.0811)
*UL*	0.0315 * (0.0186)	−0.0340 (0.0457)	−0.0025 (0.0177)	−0.0594 (0.6881)	−0.0342 (0.0677)	0.0936 (0.0711)	0.0162 ** (0.0093)	0.0020 (0.0177)	0.0182 * (0.0107)
*IS*	0.1155 *** (0.0433)	0.0021 (0.1771)	0.1176 * (0.0695)	0.0988 *** (0.0522)	0.1045 * (0.0615)	0.2033 ** (0.0968)	−0.3233 (0.2719)	0.1115 * (0.5291)	−0.2118 (0.1712)
*ES*	0.5833 ** (0.2902)	0.0732 * (0.0428)	0.6565 (1.0177)	0.1643 * (0.0923)	0.0033 (0.1078)	0.1676 * (0.0946)	0.5355 *** (0.1766)	0.0441 (0.0318)	0.5796 ** (0.3256)
*TL*	−0.0634 (0.1014)	−0.0157 *** (0.053)	−0.0791 (0.1018)	−0.0652 (0.0821)	0.0711 (0.0944)	−0.0059 (0.0512)	0.1606 (0.1003)	−0.2144 * (0.1128)	−0.0538 * (0.0302)
*EO*	0.2711 * (0.1457)	−0.1370 (02063)	0.1341 (0.1533)	0.0322 (0.2057)	−0.1069 ** (0.0523)	−0.0474 * (0.0278)	0.3770 * (0.1984)	−0.0063 (0.1030)	0.3707 * (0.2014)
*Cons.*	−0.0542 *** (0.0093)	1.0170 (1.5171)	1.0305 ** (0.4749)	0.6322 *** (0.0532)	−1.2088 *** (0.0442)	0.7080 (0.6212)	1.2023 *** (0.4322)	−0.9844 (0.8722)	1.1542 *** (0.0590)
R^2^	0.5543	0.2916	0.4677	0.5882	0.5611	0.3045	0.7421	0.6523	0.7029

Note: *, **, *** denote statistical significance at the 10%, 5%, and 1% level, respectively; the standard deviation is given in brackets.

**Table 6:** SDM regression results of one lag period of regional integration.

Variable	Direct Effect		Indirect Effect		Total Effect	
*RI* (−1)	0.4332 ***	(0.0133)	0.0928	(0.0611)	0.5260 **	(0.2182)
*UL*	−0.1644	(0.0728)	−0.0177	(0.0339)	−0.1821	(0.5263)
*IS*	0.0741 *	(0.0412)	−0.0721	(0.0802)	0.0020 *	(0.0011)
*ES*	0.3382 ***	(0.0068)	0.0318	(0.0263)	0.3700 **	(0.1762)
*TL*	−0.1022 *	(0.0568)	−0.0012	(0.0093)	−0.1034	(0.0522)
*EO*	0.1012 **	(0.5090)	−0.1521 ***	(0.0877)	0.1033 **	(0.0485)
R^2^	0.6617		0.5122		0.3842	
Wald_spatial lag	9.0493 **		13.4933 *		7.6722 *	
Wald_spatial error	5.4935 *		9.9432 *		4.9010 *	
Hausman test probability	0.0049		0.0003		0.0000	

Note: *, **, *** denote statistical significance at the 10%, 5%, and 1% level, respectively; the standard deviation is given in brackets.

## Data Availability

Not applicable.
